# Why academic selection fails to protect against physician burnout: a brief research report from a 25-year longitudinal programme

**DOI:** 10.3389/fpubh.2026.1836438

**Published:** 2026-06-02

**Authors:** Maciej Walkiewicz

**Affiliations:** Department of Psychology, Medical University of Gdańsk, Poland

**Keywords:** longitudinal study, medical education, occupational health, physician burnout, public health workforce, resilience, sense of coherence, sustainability

## Abstract

**Background:**

Physician burnout has reached crisis proportions despite increasingly competitive medical school admissions, suggesting that academic selection alone may not protect career sustainability. This report examines which baseline characteristics predict and which fail to predict long-term physician wellbeing.

**Methods:**

We report findings from a 25-year research programme at the Medical University of Gdańsk, Poland, comprising one longitudinal and two cross-sectional studies. The longitudinal study tracked the 1999 admission cohort (*n* = 320) through 6 years of medical education and 4 years post-graduation (*n* = 54). Baseline measures included academic achievement, sense of coherence (SOC-29), depression (MMPI-D), anxiety (STAI), and coping strategies (CRI). Post-graduation outcomes included licensing-examination performance (*n* = 268), career satisfaction, burnout (MBI), and quality of life. Two cross-sectional surveys (*n* = 566; *n* = 334) examined fatigue, motivation, resilience, and satisfaction.

**Results:**

Academic achievement during medical school predicted licensing-examination performance [*R*^2^ = 0.21, *F*(6,270) = 11.95, *p* < 0.001] but did not predict career satisfaction (*R*^2^ = 0.01, ns), burnout (*R*^2^ = 0.01, ns), or quality of life (*R*^2^ = 0.01–0.21, mostly ns). In contrast, sense of coherence at admission accounted for substantial variance in career satisfaction (*R*^2^ = 0.40, *p* < 0.05), general wellbeing (*R*^2^ = 0.88, *p* < 0.01), and life satisfaction (*R*^2^ = 0.40, *p* < 0.05). Cluster analysis identified three post-graduation profiles, labelled “Committed,” “Clever,” and “Bright,” differentiated by sense of coherence and coping rather than by academic achievement [*F*(2,47) = 20.18, *p* < 0.001 for satisfaction, *η^2^* = 0.46]. The most academically successful graduates (“Bright”) reported the lowest income and lowest life satisfaction, while the least academically successful (“Clever”) reported the highest. Cross-sectional surveys revealed peak study-related stress at year four (transition to clinical training) and progressive erosion of intrinsic motivation across training stages.

**Conclusion:**

Cognitive admission metrics efficiently identify students capable of mastering medical content but do not predict, and may not protect against, burnout, dissatisfaction, or impaired quality of life. We argue for reframing physician burnout as a modifiable occupational hazard and redirecting investment from selection toward resilience-building throughout medical education.

## 1 Introduction

Medical education has long assumed that rigorous academic selection produces the best physicians. This “survival of the fittest” model treats academic excellence as an indicator of professional success, including the “psychological strength” needed to withstand a demanding career (1, 2). The pre-clinical phase is widely regarded as a screening device through which only the most resilient students will pass. Yet rising rates of physician burnout, mental-health problems, and attrition across high-income countries suggest that this assumption may be incomplete (3, 4).

Despite increasingly competitive admissions processes worldwide, physician burnout has reached crisis proportions (4, 5). Depression and anxiety among medical students exceed age-matched population rates (6–8), and suicide rates among physicians remain elevated (9). The economic and human cost of physician attrition is substantial (10, 11). This is not merely an educational concern but a threat to public health. We frame physician burnout not as an individual psychological vulnerability but as an occupational health hazard rooted in a structural mismatch between selection criteria and the psychosocial demands of medical work.

The assumption that cognitive-based admissions criteria identify future physicians best equipped to sustain a demanding career has been questioned in the medical-selection literature for nearly two decades. Reviews and meta-analyses have concluded that cognitive admission metrics show only modest and inconsistent associations with downstream outcomes beyond technical examination performance (12–15). Psychological and non-cognitive characteristics, e.g., motivation, conscientiousness, coping, appear to contribute incrementally to longer-term outcomes (16, 17). Although the evidence base remains heterogeneous, and the mechanisms by which medical training shapes (or erodes) these resources are insufficiently understood.

It is against this backdrop that we present results from a 25-year research programme at the Medical University of Gdańsk, Poland. Beginning in 1999, our team has followed cohorts of medical students from admission through their professional careers, complemented in recent years by cross-sectional studies of contemporary student cohorts. The longitudinal data permit a rare opportunity to test, within one institution and using consistent measures, which baseline characteristics predict, and which fail to predict, long-term physician wellbeing.

We define career sustainability as the capacity to maintain professional competence, career satisfaction, and psychological wellbeing across the working lifespan. Drawing on this longitudinal evidence base together with two contemporary cross-sectional surveys, this Brief Research Report addresses three questions: (1) Does academic achievement predict long-term physician wellbeing, or only technical competence? (2) What psychological characteristics (measurable at admission) do predict long-term wellbeing? (3) How are these psychological characteristics distributed and changing in contemporary medical-student cohorts? The findings carry implications for medical-school selection, curricular reform, and public-health workforce policy.

## 2 Materials and methods

### 2.1 Study design and setting

This Brief Research Report integrates findings from one longitudinal cohort and two complementary cross-sectional studies, all conducted at the Medical University of Gdańsk (MUG), a major academic medical institution in northern Poland. The studies were conducted between 1999 and 2024 and have been previously published as separate empirical reports (18–25). The present report synthesises this body of work to address the three research questions specified above. Methodological details summarized here are reported more fully in the original publications. All studies received approval from the Independent Bioethics Committee of the Medical University of Gdańsk (NKBBN). All participants provided written or electronic informed consent.

### 2.2 Participants

#### 2.2.1 Longitudinal cohort (1999–2009)

The longitudinal cohort comprised all 320 students admitted to the six-year MD programme at MUG in 1999 (65% female). Of 940 candidates initially contacted before the admission examination, 365 returned baseline questionnaires (response rate 39%). Analyses were restricted to the 320 candidates who passed admission. Participants completed psychological measures annually throughout 6 years of medical training, with year-by-year retention as follows: year 0 (admission) *n* = 178, year 1 *n* = 178, year 2 *n* = 129, year 3 *n* = 127, year 4 *n* = 121, year 5 *n* = 58, year 6 *n* = 138 (18, 20–22, 24, 25). Postgraduate licensing-examination results were obtained from the Medical Examination Centre, Poland for *n* = 268 graduates (84% of the cohort). Four years after graduation (in 2009), participants were re-contacted via the Polish Chamber of Physicians; *n* = 54 (response rate 21%) provided complete outcome data. The mean age of follow-up respondents was 29.5 ± 0.8 years and 69% were female.

#### 2.2.2 Cross-sectional study 1 (2018–2019)

A cross-sectional survey of *n* = 566 s-year medical students at MUG (60% female; response rate 94%) was conducted in 2018–2019 (25). Anonymous questionnaires were distributed after psychology classes. Participation was voluntary and unincentivised.

#### 2.2.3 Cross-sectional study 2 (2024)

A multi-institutional online survey of *n* = 334 Polish medical students stratified by year of study (year 1 *n* = 119; year 4 *n* = 118; year 6 *n* = 97; 79% female; mean age 22.3 ± 2.6) was conducted in 2024 (19, 23). Participants were recruited via social media and university mailing lists.

### 2.3 Measures

Baseline measures in the longitudinal cohort included: (a) academic achievement, indexed by high-school final-examination results, the medical-school admission test (biology, chemistry, physics), and six-year grade-point averages; (b) sense of coherence, assessed by the 29-item Sense of Coherence Scale [SOC-29; (26)], measuring three components: comprehensibility, manageability, and meaningfulness; (c) depression, assessed by the Depression Scale of the Minnesota Multiphasic Personality Inventory [MMPI-D; (27); Polish adaptation (28)]; (d) trait and state anxiety, assessed by the State–Trait Anxiety Inventory [STAI; (29)); and (e) coping responses, assessed by the 8-subscale Coping Responses Inventory (CRI; (30)]. Internal consistency for these instruments has been documented in the original publications and was satisfactory in our sample.

Outcomes assessed 4 years post-graduation included: (a) postgraduate medical competence, indexed by performance on the State Examination for Medical Doctors administered by the Medical Examination Centre (a multiple-choice national licensing examination required for further specialty training); (b) career satisfaction, assessed by a three-item self-designed Cantril-scale survey (Cronbach’s *α* = 0.80); (c) burnout, assessed by the Maslach Burnout Inventory [MBI; (31); Polish adaptation (32)], with sub-scales for emotional exhaustion, depersonalisation, and personal accomplishment; and (d) quality of life, assessed by a multi-item instrument adapted from the Polish Social Diagnosis programme (33), comprising general wellbeing and health (*α* = 0.74), life satisfaction (22 items, *α* = 0.83), and self-reported income.

Cross-sectional Study 1 additionally administered the Chalder Fatigue Scale [(34); Polish validation (35)], the Schutte Self-Report Emotional Intelligence Test [SSEIT; (36, 37)], and items assessing study-related stress and satisfaction. Cross-sectional Study 2 administered the Brief Resilience Scale [BRS; (38); Polish validation (39)], self-developed motivation and satisfaction inventories, value-priority items adapted from CBOS (40), and competency-related items based on the Great Eight framework (41).

### 2.4 Statistical analysis

For the longitudinal cohort, multiple linear regression (forward selection) was used to examine predictive associations between baseline measures and post-graduation outcomes (22). Subsequently, cluster analysis (binary Euclidean distance, Ward’s method) on standardised outcome measures identified three distinct post-graduation profiles; cluster membership was further validated by discriminant analysis with backward selection (24). One-way analysis of variance with Tukey post-hoc comparisons examined differences between clusters on baseline psychological characteristics. Effect sizes are reported as *η*^2^. Missing data points were imputed by linear interpolation using existing values within the same variable, as commonly recommended for psychological longitudinal data with random missingness (42).

For cross-sectional studies, group comparisons used the Mann–Whitney *U* test (with Benjamini–Hochberg correction for multiple comparisons) and Fisher’s exact test (19). Spearman rank-order correlations examined associations between resilience, motivation, and satisfaction (23). Multiple regression examined predictors of fatigue (25). Statistical significance was set at *p* < 0.05 throughout.

## 3 Results

### 3.1 Academic achievement predicts technical competence but not career sustainability

Table 1 summarises the predictive validity of baseline measures for post-graduation outcomes. Academic achievement during medical school, indexed by six-year grade-point average, predicted performance on the postgraduate licensing examination, accounting for 21% of variance [*F*(6,270) = 11.95, *p* < 0.001]. High-school final-examination and admission-test results contributed an additional, smaller share [*R*^2^ = 0.09, *F*(7,269) = 3.87, *p* < 0.001]. However, academic achievement showed no significant association with career satisfaction [*R*^2^ = 0.01, *F*(6,48) = 0.05, ns], burnout [*R*^2^ = 0.01, *F*(6,48) = 0.09, ns], or with quality-of-life indicators [*R*^2^ = 0.01–0.21, mostly ns; (22)].

**Table 1:** Predictive validity of baseline measures (1999–2005) for postgraduate outcomes assessed 4 years after medical school graduation (longitudinal cohort, *n* varies by analysis).

Baseline measure	Licensing exam	Career satisfaction	Burnout	General wellbeing	Life satisfaction
High school + admission exam	0.09 ***	0.11 (ns)	0.10 (ns)	0.01 (ns)	0.03 (ns)
Six-year GPA	0.21 ***	0.01 (ns)	0.01 (ns)	0.15 (ns)	0.10 (ns)
Sense of coherence	0.06 (ns)	0.40 *	0.16 **	0.88 **	0.40 *
Coping strategies	0.16 (ns)	0.69 **	0.20 (ns)	0.02 (ns)	0.16 (ns)
Trait + state anxiety	0.04 (ns)	0.17 **	0.12 **	0.58 ***	0.17 **
Depression	0.03 (ns)	0.12 *	0.09 *	0.23 *	0.12 *
Need for social approval	0.01 (ns)	0.01 (ns)	0.09 *	0.24 *	0.01 (ns)
Value system	0.44 (ns)	0.40–0.50 (ns)	0.42–0.58 (ns)	0.01–0.02 (ns)	0.40–0.50 (ns)

Values are *R*^2^ (variance explained); statistical significance from *F*-tests is indicated. *R*^2^ values from multiple linear regression (forward selection). ***p < 0.001; **p < 0.01; *p < 0.05; ns = not significant. Source: Tartas et al. (22), Table 1.

In contrast, several baseline psychological characteristics predicted long-term outcomes. Sense of coherence at admission accounted for 40% of variance in career satisfaction 4 years post-graduation [*F*(11,43) = 2.60, *p* < 0.05], 16% of variance in burnout [*F*(1,54) = 10.66, *p* < 0.01], and 88% of variance in general wellbeing [*F*(12,42) = 7.03, *p* < 0.01]. Sense of coherence also predicted life satisfaction [*R*^2^ = 0.40, *p* < 0.05] and self-reported income [*R*^2^ = 0.54, *F*(2,52) = 13.17, *p* < 0.001]. Coping strategies predicted 69% of variance in career satisfaction [*F*(24,30) = 2.81, *p* < 0.01]. Trait and state anxiety predicted career satisfaction [*R*^2^ = 0.17, *p* < 0.01] and general wellbeing (*R*^2^ = 0.58, *p* < 0.001). Depression predicted career satisfaction (*R*^2^ = 0.12, *p* < 0.05) and self-reported income (*R*^2^ = 0.41, *p* < 0.01). The value-priority system, in contrast, contributed little to outcome prediction (*R*^2^ = 0.01–0.50, *F* values mostly not significant; (22)).

### 3.2 Three post-graduation profiles: cluster analysis

Cluster analysis on standardised outcome scores (postgraduate competence, career satisfaction, burnout, quality of life, income) identified three distinct post-graduation profiles among the *n* = 50 graduates with complete outcome data (20, 21, 24). The clusters differed significantly across all outcome domains (Table 2).

**Table 2:** Three post-graduation profiles identified by cluster analysis: outcome scores (longitudinal cohort, *n* = 50).

Outcome	Committed (*n* = 14)	Clever (*n* = 20)	Bright (*n* = 16)	*F*(2,47), *p*, *η*^2^
Licensing exam (*z*)	−0.62 ± 0.61	−0.50 ± 0.53	+1.17 ± 0.61	48.26, *p* < 0.001, *η*^2^ = 0.53
Career satisfaction (*z*)	+0.95 ± 0.68	−0.47 ± 0.47	−0.24 ± 0.85	20.18, *p* < 0.001, *η*^2^ = 0.46
Burnout (*z*)	+0.33 ± 0.48	−0.27 ± 0.43	+0.07 ± 0.45	7.37, *p* < 0.001, *η*^2^ = 0.24
General wellbeing (*z*)	−0.29 ± 0.81	+0.49 ± 0.55	−0.35 ± 0.60	9.50, *p* < 0.001, *η*^2^ = 0.29
Life satisfaction (*z*)	−0.60 ± 0.78	+0.76 ± 0.45	−0.89 ± 0.86	29.09, *p* < 0.001, *η*^2^ = 0.55
Income (1–4 scale)	3.57 ± 0.51	3.80 ± 0.41	2.63 ± 0.89	16.80, *p* < 0.001, *η*^2^ = 0.42

Values are M ± SD (*z*-scores for standardised outcomes; raw scale for income). Cluster analysis used binary Euclidean distance and Ward’s method on *z*-standardised outcome scores. Source: Walkiewicz et al. (24), Table 1.

The first cluster (*n* = 14, 28%), labelled the “Committed,” reported the highest career satisfaction (*z* = +0.95) but the highest burnout (*z* = +0.33) and below-average licensing-examination performance (*z* = −0.62). The second cluster (*n* = 20, 40%), labelled the “Clever,” reported below-average career satisfaction (*z* = −0.47) but the lowest burnout (*z* = −0.27), the highest general wellbeing (*z* = +0.49), the highest life satisfaction (*z* = +0.76), and the highest self-reported income (M = 3.80 on a 4-point scale). The third cluster (*n* = 16, 32%), labelled the “Bright,” was characterised by the highest licensing-examination performance (*z* = +1.17) but reported below-average career satisfaction (*z* = −0.24), the lowest general wellbeing (*z* = −0.35), the lowest life satisfaction (*z* = −0.89), and the lowest self-reported income (*M* = 2.63). Differences between clusters were substantial in size [satisfaction: *F*(2,47) = 20.18, *p* < 0.001, *η*^2^ = 0.46; life satisfaction: *F*(2,47) = 29.09, *p* < 0.001, *η*^2^ = 0.55; income: *F*(2,47) = 16.80, *p* < 0.001, *η*^2^ = 0.42].

Cluster membership could be partially anticipated from baseline psychological characteristics. The “Clever” displayed the highest baseline sense of coherence at admission [M = 145.0 ± 21.9 vs. M = 125.0 ± 25.5 for “Committed” and M = 125.6 ± 20.7 for “Bright”; *F*(2,47) = 4.54, *p* < 0.05, *η*^2^ = 0.19; (20)]. Differences in SOC components, particularly comprehensibility and manageability, persisted throughout medical school [year 1 comprehensibility: *F*(2,47) = 11.03, *p* < 0.001, *η*^2^ = 0.47; year 5 manageability: *F*(2,47) = 6.49, *p* < 0.01, *η*^2^ = 0.28]. Trait anxiety differentiated clusters from year two onwards, with the “Clever” consistently displaying the lowest scores [year 5 trait anxiety: *F*(2,47) = 15.37, *p* < 0.001, *η*^2^ = 0.65; (24)]. Coping strategies further differentiated the “Clever”: at admission, they exhibited the highest problem-solving coping [*F*(2,47) = 4.38, *p* < 0.05] and the lowest acceptance/resignation [*F*(2,47) = 6.86, *p* < 0.01] and emotional discharge [*F*(2,47) = 4.49, *p* < 0.05; (21)]. In contrast, six-year grade-point averages did not differentiate clusters (*F* values mostly not significant), confirming that academic achievement alone could not distinguish among the three post-graduation trajectories.

### 3.3 Contemporary student experience: cross-sectional findings

Two cross-sectional studies of more recent cohorts examined how psychological resources and study-related strain are distributed across stages of training. In Cross-sectional Study 1 (*n* = 566 s-year students), a multiple-regression model accounted for 28% of variance in self-reported fatigue (Chalder Fatigue Scale; (25)). Significant predictors were study-related stress (*β* = +0.31, *p* < 0.001), study satisfaction (*β* = −0.18, *p* < 0.001), the “using emotions” component of emotional intelligence (*β* = −0.13, *p* = 0.005), comprehensibility component of sense of coherence (*β* = −0.12, *p* = 0.002), quality of life (*β* = −0.10, *p* = 0.02), and counter-intuitively, the “recognising emotions” component of emotional intelligence (*β* = +0.08, *p* = 0.04).

In Cross-sectional Study 2 (*n* = 334 students stratified by year), self-reported study-related stress peaked at year 4 (median 5, IQR 3–6 on a 7-point scale), significantly higher than at year 1 (median 3, IQR 2–5; *U* = 4,613, *p* < 0.001) and year 6 [median 3, IQR 2–5; *U* = 7,962.5, *p* < 0.001; (19)]. Overall satisfaction with medical studies declined progressively across years (year 1 median 6, year 4 median 5, year 6 median 5; Y1 vs. Y6: *U* = 8,668, *p* < 0.001). Intrinsic motivation (“my interests”) was rated significantly higher at year 1 than year 6 (median 7 vs. 6; *U* = 6,942, *p* < 0.05); extrinsic motivation (“high income”) declined between year 1 and year 4 (median 6 vs. 5; *U* = 8,396, *p* < 0.05). Resilience scores did not differ significantly across years.

Resilience showed consistent positive associations with multiple satisfaction domains in Cross-sectional Study 2 (Spearman *ρ* = 0.20–0.29, all *p* < 0.001 across satisfaction sub-scales; (23)). Students who reported lasting effects of the COVID-19 pandemic, perceived knowledge gaps, reduced practical skills, or diminished confidence as future physicians, showed lower satisfaction across multiple domains compared to peers without such concerns (e.g., overall satisfaction: *U* = 13,655, *p* < 0.001 for those reporting reduced confidence).

## 4 Discussion

### 4.1 Principal findings

These results from longitudinal and two contemporary cross-sectional studies, converge on three principal findings.

1. Academic achievement during medical school is predictive of technical competence on the postgraduate licensing examination but does not predict long-term career satisfaction, burnout vulnerability, or quality of life.
2. Several psychological characteristics measurable at admission, most prominently sense of coherence, but also coping strategies, anxiety, and depression, show substantial predictive associations with these long-term outcomes.
3. Contemporary cross-sectional data suggest that the psychological resources protective against burnout are not stable. Intrinsic motivation declines across training stages, study-related stress peaks during the transition to clinical training, and recent disruptions (such as the COVID-19 pandemic) compound these strains.

A particularly counter-intuitive result merits emphasis. Among the three post-graduation profiles identified by cluster analysis, the most academically successful graduates (the “Bright”) reported the lowest income and the lowest life satisfaction, whereas the least academically successful (“Clever”) reported the highest of both. This pattern challenges the implicit assumption that academic excellence confers a “head start” across different dimensions of professional life.

### 4.2 Comparison with the existing literature

Our finding that cognitive admission metrics show modest predictive value beyond technical competence is consistent with international evidence. Reviews and meta-analyses (12–15) have consistently found that academic measures explain only a small share of variance in non-cognitive outcomes such as professionalism, communication, or career satisfaction. Longitudinal studies from Norway (43), the United Kingdom (44), and the United States (45) have converged on the same conclusion. Our data add a Polish, Central European voice to this evidence base and document the same pattern over a 25-year horizon.

The predictive role of sense of coherence in our cohort is consistent with the broader salutogenic literature (46), which has documented robust associations between SOC and health outcomes across diverse populations. Our findings extend this evidence to physician career sustainability specifically. Similarly, the protective role of task-oriented coping and the risk associated with emotion-oriented coping align with prior systematic reviews of physician and medical-student samples. The decline in intrinsic motivation observed in our cross-sectional data is consistent with self-determination theory (47, 48) and with U.S. cohort studies documenting declining wellbeing across training stages (49).

The progressive trajectory captured in our cross-sectional data: declining intrinsic motivation, persistent (untapped) resilience, and peak stress at the preclinical-clinical transition, also parallels broader generational trends in mental-health indicators among young adults (50). Whether these reflect intrinsic generational shifts, contextual factors specific to medical training, or interactions between the two cannot be resolved from our data; this question warrants further longitudinal cohort comparison.

### 4.3 Implications for medical education and workforce policy

These findings carry implications at two levels: institutional practice and public-health workforce policy. At the institutional level, our data suggest that wellbeing is better understood as a support problem than a selection problem. Academic selection ensures competence but does not prevent burnout. We recommend four directions for educational reform: (A) Confidential self-assessment of psychological resources early in training, designed to build metacognitive awareness rather than to gatekeep. (B) Universal, curriculum-integrated coping-skills training for all students, framed as professional development (e.g., “performance psychology”) rather than remedial therapy (51). (C) Systematic longitudinal monitoring of student wellbeing throughout education, as recommended in recent expert consensus on physician burnout prevention (52). (4) Structural reform of educational practices that function as psychosocial hazards. Transition from numerical grading to pass/fail assessment in pre-clinical years, which has been shown to reduce stress without compromising academic performance (53). Reform of the hidden curriculum that teaches emotional suppression as a marker of professionalism (54).

At the public-health workforce level, the cross-sectional findings (Table 3) point to specific targets for these interventions. The preclinical-to-clinical transition as the critical intervention point, intrinsic motivation as the resource most vulnerable to erosion, and resilience as an untapped developmental asset. The proposed interventions are not aimed at “toughening up” students but at challenging educational practices that undermine meaningfulness, predictability, and agency. If the system of medical education consistently produces physicians who are competent yet psychologically fragile, then burnout is not a “side effect” of that system; it is its logical consequence.

**Table 3:** Selected cross-sectional findings: stress, motivation, and satisfaction across stages of medical training (cross-sectional study 2, *n* = 334).

Variable	Year 1 (*n* = 119)	Year 4 (*n* = 118)	Year 6 (*n* = 97)	Mann–Whitney *U*, *p*
Stress level (median, IQR)	3 (2–5)	5 (3–6)	3 (2–5)	Y1 vs. Y4: *U* = 4,613, *p* < 0.001; Y4 vs. Y6: *U* = 7,962.5, *p* < 0.001
Overall satisfaction with studies	6 (5–6.5)	5 (3–6)	5 (2–6)	Y1 vs. Y6: *U* = 8,668, *p* < 0.001; Y4 vs. Y6: *U* = 7,233.5, *p* < 0.001
Satisfaction with current university	6 (6–7)	6 (5–7)	5 (3–6)	Y1 vs. Y6: *U* = 8,505, *p* < 0.001; Y4 vs. Y6: *U* = 7,199, *p* < 0.001
Motivation: my interests	7 (6–7)	– (ns)	6 (5–7)	Y1 vs. Y6: *U* = 6,942, *p* < 0.05
Motivation: high income	6 (5–7)	5 (5–6)	– (ns)	Y1 vs. Y4: *U* = 8,396, *p* < 0.05
Resilience (BRS)	– (ns)	– (ns)	– (ns)	No significant differences

Median (IQR) on 7-point scales for stress, satisfaction, and motivation. Benjamini–Hochberg correction applied. Source: Sarnowska et al. (19), Tables 1, 2, and 6.

### 4.4 Strengths and limitations

The principal strength of this report is the unusual time horizon. A single institution followed across 25 years, with consistent measurement and a clearly defined cohort. The integration of longitudinal and contemporary cross-sectional data permits triangulation across two generations of medical students.

Several limitations should be acknowledged. First, the principal findings originate from a single national and institutional context, which limits generalisability. Replication in other settings, particularly outside Central Europe, is needed. Second, the four-year post-graduation follow-up of the longitudinal cohort achieved a response rate of 21% (*n* = 54 of 255 reachable graduates), and cluster analyses are based on *n* = 50. This places caveats on the precision of point estimates and on the stability of cluster solutions. However, the convergence of findings across multiple statistical approaches and across publications adds confidence. Third, attrition between baseline and follow-up may have introduced selection bias if non-respondents differed systematically on baseline characteristics. Fourth, although we interpret psychological characteristics as predictive of long-term outcomes, the design is correlational. We cannot establish causal relationships, and unmeasured variables (e.g., specialty choice, workplace conditions) likely contribute to the observed associations. Fifth, the cross-sectional studies cannot speak to within-person change over time. Sixth, cohort comparisons over time inevitably reflect broader social and educational changes. The contemporary cross-sectional data describe contemporary cohorts but cannot establish whether the patterns observed represent generational change. Finally, this report integrates findings previously published as separate empirical studies. It does not present new analyses, and readers seeking full statistical detail are referred to the original publications.

## 5 Conclusion

Cognitive admission metrics efficiently identify students capable of mastering medical content, but they do not predict and may not protect against burnout, dissatisfaction, or impaired quality of life. The psychological characteristics that do predict long-term physician wellbeing are rarely assessed during selection, often eroded during training, and appear under additional strain in contemporary cohorts. We propose that this pattern be reframed. Physician burnout is not a side effect of medical practice, but a foreseeable product of a selection-and-training system optimised for short-term technical performance rather than long-term occupational sustainability.

The solution is not to abandon academic selection but to expand the conception of educational responsibility. We must shift from the question “Who is suited to be a physician?” to “How do we help physicians remain fit throughout their careers?” This requires redirecting investment from selection toward systematic support and resilience-building throughout medical education, a reframing of physician burnout as a modifiable occupational hazard rather than an individual vulnerability.

## Data Availability

The data analyzed in this study is subject to the following licenses/restrictions: the original contributions presented in this report are included in the article. The primary empirical data underlying the findings summarized here are available in the original publications cited throughout the text. The raw datasets are not publicly available due to participant privacy considerations but are available from the corresponding author upon reasonable request. Requests to access these datasets should be directed to maciej.walkiewicz@gumed.edu.pl.
